# Effect of including n-3/n-6 fatty acid feed sources in diet on fertility and hatchability of broiler breeders and post-hatch performance and carcass parameters of progeny

**DOI:** 10.5713/ajas.19.0055

**Published:** 2019-04-15

**Authors:** Seyyed Naeim Saber, Hasan Rustu Kutlu

**Affiliations:** 1 Animal Science Department, Faculty of Agriculture, Cukurova University, Adana, 1330, Turkey

**Keywords:** Breeder, n-3/n-6 Fatty Acid, Hatchability, Progeny

## Abstract

**Objective:**

The present trial was conducted to determine the influence of different dietary fatty acid (omega-3 and omega-6) sources on reproductive performance of female broiler breeders and growth performance and carcass traits of their progeny.

**Methods:**

Two hundred and twenty, 25 weeks old Ross-308 male (20) and female (200) broiler breeders were used in the experiment for the period of 6 weeks. All birds were randomly divided into four dietary treatments (containing 2% soybean oil, 2% sunflower oil, 2% flaxseed oil, and 2% fish oil) each with five replicates of one male and ten females. Throughout this experiment hatching performance of broiler breeders, progeny growth performance and carcass parameters were recorded.

**Results:**

The results showed that the inclusion of different fatty acid sources in female broiler breeders diet had no significant effects (p>0.05) on number of fertile eggs, post-hatch mortality, and fertility rate. The soybean oil supplemented group had significantly (p<0.05) higher late embryonic mortality compared to other three treatments.

**Conclusion:**

It was concluded that inclusion of 2% of different sources of omega-3 and omega-6 fatty acids (especially 2% flax seed oil) in broiler breeders’ diet can reduce late embryonic mortality. The other reproductive characteristics of parents and growth and carcass characteristics of progeny remained unaltered by dietary sources of omega-3 and omega-6 fatty acids.

## INTRODUCTION

It is well recognized that hatchability and female reproductive performance are the most important factors in poultry production. In poultry industry, some parameters such as hatchability and abdominal fat deposition [1], fatty acid profile of egg yolk [2,3], and lipid metabolism in chicks [4] can be influenced by the ratio of dietary omega-3 to omega-6 fatty acids, as the commercial diets used in breeders are formulated in favor of omega-6. However, it has been reported that the absolute amount of n-3 and n-6 fatty acids in broiler breeder diet may be more important than n-6/n-3 fatty acid ratios and should be considered in the reproductive and performance traits of breeders. Therefore, it is necessary to supply higher levels of n-3 and n-6 fatty acid with respect to n-6/n-3 fatty acid ratios [5]. It is hypothesized that inclusion of omega-3 and omega-6 in broiler breeders’ diet could potentially improve incubation parameters and progeny performance by improving egg yolks’ fatty acid profile and transitioning these fatty acids from egg to embryonic tissues. It has long been known that egg yolk is the primary energy source for embryos during their development, whose nutrient profile changes with the maternal diet [6,7] and thereby differences in yolk would create differences in the nutritional status of progeny [8]. Some studies reported that normal embryonic growth and development depend on a complete supply of nutrients within the egg and it can influence hatchability, fertility, progeny growth, immunity, and carcass characteristics [9–11]. Various research findings have indicated that one of those nutrients is omega-3 fatty acid but the present literature presents conflicting results; it has been reported that diets enriched with omega-3 polyunsaturated fatty acid have an improving effect on immunity [12] but another study reported no beneficial effects of omega-3 fatty acid [13] or a detrimental effect on chickens’ immunity [14]. It should also be noted that yolk omega-3 fatty acid has an unique role in eicosanoid metabolism and modulation of progeny as omega-3 fatty acid is taken up from yolk sac lipids and incorporated into cell membrane phospholipids of the developing embryo during avian embryo genesis and post hatch growth [3,4]. It has been reported that during embryo development fatty acid from the maternal diet incorporated in the yolk is transferred into liver of offspring through the yolk residual by the time of hatching [3]. Moreover, in our previous experiment, we observed that dietary inclusion of 2% flaxseed oil as a source of omega-3 had a potential to improve laying performance, egg quality parameters, and egg yolk fatty acid composition in broiler breeders [2]. The present study was, therefore, aimed to investigate the effects of dietary inclusion of omega-3 fatty acids from flaxseed oil and/or fish oil and omega-6 fatty acids from soya oil in broiler breeders’ diet on hatchability, fertility, and post-hatch growth performance and carcass parameters of progeny.

## MATERIALS AND METHODS

### General experimental procedure and trial groups

The present study was carried out in the Broiler Unit of Experimental Farm of the Department of Animal Science, Faculty of Agriculture, University of Cukurova-Turkey. All the protocols used in this experiment were approved by the Animal Experiments Local Ethics Committee of Cukurova University, Adana-Turkey.

Two hundred and twenty, 25 weeks old Ross-308 (200 female and 20 male) broilers were used in this experiment. During pre-feeding period (25 and 26th weeks), all broiler breeders were fed with standard breeder diet based on corn and soybean. After 2 weeks of pre-feeding period all female birds were divided according to body weight and egg production in a completely randomized design into four experimental diets (Table 1) containing 2% soybean oil or 2% sunflower oil or 2% flax seed oil or 2% fish oil groups with five replicate pens for each treatment and therefore total 20 replicates, each containing ten females and one male. All male broiler breeders were fed with standard breeder diet based on corn and soybean (pre-feeding period diet). Fatty acids composition of oil sources used in this experiment is shown in Table 2. The trial lasted for 6 weeks (27 to 32th weeks of age). Each pen (sizing 2×1.5 meters) of the breeder unit had five nests bedded with wood shaving, sized 25×43×35 cm each, a female tubular feeder and one individual male feeder and nipple drinkers. The birds were placed on wood shaving litter 7 to 8 cm height. The lighting (16:8 light:dark photoperiod) and feed (female: 163 g/d, male: 130 g/d) were provided according to the recommendation of Ross Breeding Company [15] with *ad libitum* access to drinking water. The environmental temperature (18°C to 22°C) and humidity (55% to 60% relative humidity [RH]) were maintained within the animal comfort zone using foggers and tunnel ventilation. During the experiment, males’ performances were observed and the males with low mating performance were replaced with spare males.

### Fertility and hatching performance

At the 30th week of age, eggs were checked and collected for fertility testing (with bright light) and to understand male broiler breeders reproductive performance before incubation. The hatching capacity of the eggs, obtained from the broiler breeders, receiving different sources of omega-3 and omega-6 fatty acids was examined in the 21st day of incubation. For this purpose, at 31st and 32nd weeks of age, the eggs with double yolk, dirty eggs, thin shell eggs, broken eggs, and cracked eggs were removed and the remaining ones stored in a cool room (12°C with 70 RH) until we obtained 700 eggs (35 for each replicate). The eggs were incubated in a single stage incubator and temperature was controlled according to McQuoid [16]. At the end of 21st day of incubation, all hatched chicks were carefully taken from the pouches and weighted by an electronic scale with a sensitivity of 0.01 g. After weighting, the chicks were sexed by wing feathers according to Ross (Aviagen) [17] recommendation. The eggs that failed to hatch were broken to determine the cause and to classify as infertile or dead embryos to calculate hatchability of total as well as fertile eggs. A visual estimation of the age at death was carefully performed and embryo mortality was separated as early, mid and late dead in shell (1 to 7 days; 8 to 14 days; 15 to 21 days). The percentage of hatching chicks considered unsuitable for placement as well as pips was calculated. The difference between total eggs set and infertile eggs allowed the calculation of the present hatchability of fertile eggs. So, fertility rate (%), total hatchability (%), and hatchability of fertile eggs (%) were calculated [18] as given below;

$$\begin{array}{l} \text{Fertility rate }(\%)=(\text{no of fertile eggs}/\text{no of eggs placed in hatchery})\times 100\\ \text{Total hatchability }(\%)=(\text{no of chicks hatched}/\text{no of eggs placed in hatchery})\times 100\\ \text{Hatchability of fertile eggs }(\%)=(\text{no of chicks hatched}/\text{no of fertile eggs placed in hatchery})\times 100 \end{array}$$

### Growth performance of progeny

At the end of the incubation, all chicks obtained from the eggs were carefully taken and divided into 20 pens by the maternal group/subgroup number to follow on the same axis as the maternal to its chicks. Each treatment group had 5 pens sized 2×3 m that were equipped with a tube feeder and an automatic water-bowl on litter; wood shaving litter 7 to 8 cm height. During the experiment, all chicks were fed with starter (0 to 10 days of age), grower (11 to 21 days of age), finisher (22 to 29 days of age), and withdrawal (30 to 35 days of age) diets formulated as per Ross recommendations [17]. Chicks were fed for 35 days *ad libitum* under a 23:1 light:dark photoperiod. Ingredient and nutritional compositions of the diet are given in Table 3.

The production parameters like live weight, feed intake and feed efficiency were recorded weekly during the experiment. At 35th day of age, all the birds were subjected to slaughter studies.

### Carcass parameters of progeny

At the end of the experiment (35 days of age), all chicks were weighted and 6 birds (3 male+3 female) from each subgroup were selected and slaughtered according to the average body weight of groups in order to determine the hot carcass weight, abdominal fat, and carcass yield.

### Statistical analysis

The data obtained in the study were analysed using general linear model procedure of the Statistical Analysis System [19] to obtain the effect of oil source. Duncan’s New Multiple Range Test in SAS was used to identify significant differences among treatments means [19]. Results obtained in this study were presented as means per bird with standard errors of the difference between means (SED; $\sqrt{\frac{s^{2}}{\text{n}}}$) with p values, except for feed intake of breeders as feeds were given to the them in equal amounts according to the recommendation of the Breeding Company [17].

## RESULTS

### Fertility and hatching performance

The effects of dietary inclusion of omega-3 and omega-6 fatty acids sources on hatchability and fertility parameters in broiler breeders are given in Table 4. The dietary omega-3 and omega-6 fatty acids did not have a significant (p<0.05) effect on number of fertile eggs, embryonic mortality in early stage, embryonic mortality in mid stage, post-hatch mortality and fertility rate. However, results of the present experiment showed that the inclusion of different sources of omega-3 and omega-6 fatty acids in breeder diets had a significant (p<0.05) effect on late stage embryo mortality, number of chicks hatched alive, chicks’ weight at hatching, hatchability of fertile eggs and total hatchability.

### Growth performance of progeny

The effects of different dietary fatty acid sources in maternal diet on progeny performance were obtained as shown in Table 5. On perusal of the results of weekly feed intake of broiler chickens, it was seen that the inclusion of different dietary sources of omega-3 and omega-6 fatty acids in their parents’ diets had no significant effect on production performance of broiler progeny.

### Carcass parameters of progeny

The effects of the inclusion of different fatty acid sources in maternal diet on carcass parameters of progeny were analyzed as shown in Table 6. There were no significant effects on body weight parameter between experimental groups (p> 0.05). Inclusion of 2% different fatty acids in broiler breeders’ diet did not affect any of the carcass traits (hot carcass weight, abdominal fat, and carcass yield) studied in this experiment.

## DISCUSSION

The results obtained in the trial suggest that inclusion of omega-3 and omega-6 fatty acid sources in broiler breeders’ diets had significant effect on incubation parameters. The results of this experiment are in agreement with the findings of Al-Daraji et al [20] who found that adding of 3% corn, fish, flax, and sunflower oil in quails’ diet had a significant effect on fertility, hatchability of total eggs, hatchability of fertility eggs, and embryonic mortality. Also Bozkurt et al [10] suggested that adding of herbal essential mixture oil in broiler breeders’ diet had a significant effect on fertility, hatchability of total eggs, chick weight, and total chicks (p<0.05). But our experiment results are in disagreement with the finding of Koppenol et al [4], who reported that supplementation of broiler breeders’ diet with DHA and EPA does not have significant effect on incubation parameters (p>0.05). Also Pirsarayi et al [21] suggested that adding of different levels of tallow in broiler breeders’ diet did not have a significant effect on livability, fertility, and hatchability. It was also reported that laying quails’ diet containing flax seed oil could improve the productive traits and this might be attributed to hormone metabolism regulation, mainly estrogen [22]. In another study, it was reported that yolk fat is a primary and important source of energy and essential nutrient for embryonic development [7, 23]. It has been hypothesized that some fatty acids such as eicosapentaenoic acid and docosahexenoic acid available in yolk are essential for embryonic development and they can improve incubation parameters such as chicks’ quality and prevent embryo mortality in different stages of embryo development. In the present study, it was shown that the inclusion of omega fatty acids can prevent embryonic mortality in late stage.

Inclusion of 2% different omega-3 and omega-6 fatty acids in broiler breeders’ diet had a significant effect on progeny feed intake and feed conversion ratio at the 7th and 14th day of age but this difference disappeared after 15th day of age. When the body weight parameter is investigated, it can be seen that there is not a significant difference between progeny during the experiment. Speake et al [24] reported that liver is a main organ for lipid metabolism and fatty acids from the yolk are transferred via the portal system to the developing liver of embryo. On the other hand, Wilson [25] explained that the eggs with large size have larger chicks and this advantage continues into market age. In this experiment larger eggs obtained from broiler breeders which received diets containing sunflower oil, fish oil, and fish oil dietary oils which led to the production of heavy chicks. Therefore, the existing difference (p<0.05) in feed conversion ratio at the 7th and 14th days of age is related to the chicks’ weight at hatching time. So, it can be said that inclusion of different sources of oil maternal diets does not affect post-hatch feed intake and body weight. The results obtained from this study were in line with Pappas et al [26,7] studies. They reported that supplementation of broiler breeders’ with eicosapentaenoic acid caused a lower daily weight gain of the progeny during starter period and this supplementation did not have a significant effect on feed intake in progeny.

In this study, there were not any significant effect on the average of hot carcass weight, abdominal fat, and carcass yield. This experiment results are disagreement with the finding of Kidd et al [27]. They stated that manipulation of breeders’ diet with L-carnitine (25 mg/kg) had a significant effect on carcass fat and breast meat in male and female progeny.

## CONCLUSION

The results obtained from this study showed that inclusion of 2% of different sources of omega-3 and omega-6 fatty acids (especially 2% flax seed oil) in broiler breeders’ diet can influence late embryonic mortality with 2% sun flower oil supplementation having significantly (p<0.05) higher value compared to other treatments. The other reproductive characteristics of parents and growth and carcass characters of progeny remained unaltered due to different dietary sources of omega-3 and omega-6 fatty acids.

## Figures and Tables

**Table 1:** Composition and calculated analysis of the basal diet given female breeders

Ingredients (%)	Dietary treatments				Nutrient composition (%)	

	SO	SA	FL	FI		
Yellow corn	54.49	54.49	54.49	54.49	Dry matter	88.52
Soybean meal	10.00	10.00	10.00	10.00	Crude protein (N×6.25)	19.00
Fullfat soybean	9.64	9.64	9.64	9.64	Crude fiber	3.58
Limestone (GRN)	7.71	7.71	7.71	7.71	Crude fat	3.71
Sunflower meal-36	7.46	7.46	7.46	7.46	Crude ash	13.35
Corn gluten meal-60	3.86	3.86	3.86	3.86	Starch	34.99
Meat-Bone35	2.48	2.48	2.48	2.48	Metabolizable energy (poultry, kcal/kg)	2,680
DCP-18	1.57	1.57	1.57	1.57	Ca	3.65
Soybean oil	2	-	-	-	Tot. P	0.78
Sunflower oil	-	2	-	-	Ava-P	0.50
Flax seed oil	-	-	2	-	Na	0.16
Fish oil	-	-	-	2	Lysine	0.87
Salt	0.24	0.24	0.24	0.24	Methionine	0.37
Vitamin premix1)	0.20	0.20	0.20	0.20	Methionine+cystine	0.70
Mineral premix2)	0.10	0.10	0.10	0.10	Tryptophan	0.20
Sodium bicarbonate	0.10	0.10	0.10	0.10	Threonine	0.70
L-lysine	0.06	0.06	0.06	0.06	Arginine	1.20
Choline-60	0.05	0.05	0.05	0.05	Isoleucine	0.76
DL-methionine	0.04	0.04	0.04	0.04	Valine	0.89
Total	100	100	100	100		

SO, 2% soybean oil; SA, 2% sunflower oil; FL, 2% flaxseed oil; FI, 2% fish oil.

1) Vitamin premix (per 2 kg of diet): vitamin A, 16,000 IU; vitamin D_3_, 3,000 IU; vitamin E, 40 IU; vitamin K_3_, 2.5 mg; vitamin B_1_, 2.5 mg; vitamin B_2_, 10 mg; nicotinamide, 50 mg; calcium D-pantothenate, 15 mg; vitamin B_6_, 6.25 mg; vitamin B_12_, 0.035 mg; folic acid, 15 mg; D-biotin, 0.045 mg; choline chloride, 150 mg.

2) Mineral premix (mg/kg of diet): Mn, 80; Fe, 80; Zn, 60; Cu, 8; Co, 0.2; I, 0.5; Se, 0.15.

**Table 2:** Fatty acids composition of oil sources used in broiler breeder diets (%)

Numeric name	Common name	Oil sources			

		SO	SA	FL	FI
C12:0	Lauric asit	0.06	0.05	0.03	0.03
C14:0	Myristic acid	-	-	-	3.90
C14:1	Miristoleic acid	-	-	-	0.14
C16:0	Palmitic acid	10.05	5.79	5.94	12.50
C16:1	Palmitoleic acid	0.34	0.24	0.24	4.28
C17:0	Heptadecanoic acid	0.05	-	-	0.17
C17:1	cis-17-heptadecenoic acid	0.04	-	-	0.16
C18:0	Stearic acid	4.56	3.88	4.36	3.80
C18:1 n9	oleic acid	23.54	34.98	24.53	28.57
C18:2 n6	Linoleic acid	52.80	53.47	14.68	11.21
C18:3 n3	Linolenic acid	7.68	0.22	49.87	4.14
C20:0	Arachidic acid	-	-	-	0.79
C20:1	cis-11-Eicosenoic acid	-	-	-	0.18
C20:2	cis-11,14-Eicosadienoic acid	-	-	-	0.37
C20:3 n6	cis-8,11,14-Eicosatrienoic acid	-	-	-	0.72
C20:4 n6	Arachidonic acid	-	-	-	4.95
C20:5 n3 (EPA)	cis-5,8,11,14,17-eicosapentaenoic acid	-	-	-	3.24
C22:2	cis-13,16-Docosadienoic acid	-	-	-	0.18
C22:6 n3 (DHA)	cis-4,7,10,13,16,19-Docosahexaenoic acid	-	-	-	8.53
Total of omega-3 fatty acids		7.68	0.22	49.87	15.91
Total of omega-6 fatty acids		52.80	53.47	14.68	16.88
Total of saturated fatty acids		14.72	9.72	10.32	21.19
Total of mono unsaturated fatty acids		23.92	35.22	24.77	33.33
Total of polyunsaturated fatty acids		60.48	53.69	64.54	32.98

SO, soybean oil; SA, sunflower oil; FL, flax oil; FI, fish oil.

**Table 3:** Ingredient and nutrient compositions of broiler diets

Ingredients (%)	Starter (0 to 10 days)	Grower (11 to 21 days)	Finisher (22 to 29 days)	Withdrawal (30 to 35 days)
Yellow corn	43.17	46.63	50.70	50.76
Soybean meal (47.5% CP)	15.64	7.71	-	-
Full fat soya	14.17	16.68	26.21	26.21
Wheat short (15% CP)	13.03	13.00	11.17	11.17
Maize gluten meal (60% CP)	5.00	3.00	-	-
Poultry offal meal (52% CP)	-	4.00	4.00	4.00
Meat-Bone meal (33% CP)	4.00	5.27	4.49	4.49
Soya oil	2.00	2.00	2.00	2.00
DCP (18% P)	0.60	-	-	-
Sodium bicarbonate	0.11	0.08	-	-
Common salt	0.17	0.14	0.21	0.21
Bio-lysine (60%)	0.77	0.60	0.36	0.36
Limestone	0.61	0.28	0.26	0.26
DL-methionine	0.36	0.25	0.24	0.24
Anticoccidial	0.06	0.06	0.06	-
Vitamin premix1)	0.20	0.20	0.20	0.20
Mineral premix2)	0.10	0.10	0.10	0.10
Total	100.00	100.00	100.00	100.00
Nutrient composition (%)				
Dry matter	88.00	88.00	88.00	88.00
Crude protein	24.00	22.00	21.00	20.00
Ether extract	7.00	8.66	10.13	10.13
Crude fibre	3.20	3.17	3.37	3.37
Crude ash	6.03	5.80	5.48	5.48
Lysine	1.43	1.26	1.09	1.09
Methionine	0.70	0.56	0.50	0.50
Methionine+cystine	1.07	0.84	0.86	0.86
Calcium	1.00	1.00	0.90	0.90
Available phosphor	0.45	0.45	0.40	0.40
Sodium	0.16	0.16	0.16	0.16
Metabolizable energy (kcal/kg)	3,050	3,150	3,250	3,250

CP, crude protein; DCP, dicalcium phosphate.

1) Each 2 kg of vitamin premix contains: 15,500,000 IU vitamin A; 5,000,000 IU vitamin D_3_; 100,000 mg vitamin E; 3,000 mg vitamin K_3_; 3,000 mg vitamin B_1_; 8,000 mg vitamin B_2_; 60,000 mg niacin; 15,000 mg Ca-D-Pantotenate; 5,000 mg vitamin B_6_; 20 mg vitamin B_12_; 2,000 mg folic acid; 200 mg D-biotin; and 100,000 mg vitamin C.

2) Each kg of trace mineral premix contains: 80,000 mg manganese; 60,000 mg iron; 60,000 mg zinc; 5,000 mg copper; 200 mg cobalt; 1,000 mg iodine; 200 mg selenium (sodium selenite); 500,000 choline chloride.

**Table 4:** The effect of dietary fatty acid (n-3 and n-6) sources on hatching performance in broiler breeders

Parameters	Dietary fat sources				SED	p-value

	SO	SA	FL	FI		
Number of eggs placed hatchery (No/replication)	35	35	35	35	-	-
Number of fertile eggs (number/replication)	34.40	33.60	34.60	34.40	0.18	0.263
Embryonic mortality in early stage	1.00	1.80	0.80	1.80	0.23	0.340
Embryonic mortality in mid stage	0.00	0.80	0.40	0.40	0.15	0.346
Embryonic mortality in late stage	4.40^a^	1.60^b^	0.40^b^	1.60^b^	0.32	0.003
Number of chicks hatched alive	28.00	29.20	32.00	30.20	0.56	0.121
Male (number)	15.20	12.40	14.00	15.00	χ^2^** = 5.25	0.154
Female (number)	12.80	16.80	18.00	15.20		
Chicks’ weight at hatching (g/chick)	48.85	50.81	53.60	53.01	0.66	0.082
Post-hatch mortality (number)	0.80	0.20	0.20	0.20	0.10	0.121
Crippled chicks (number)	0.20	0.00	0.80	0.20	0.11	0.130
Fertility rate (%)	98.29	96.00	98.86	98.28	0.52	0.263
Hatchability of fertile eggs (%)	84.30	87.62	95.36	88.86	1.44	0.090
Total hatchability (%)	82.86^b^	84.00^b^	94.29^a^	87.43^ab^	1.50	0.065

SO, 2% soybean oil; SA, 2% sunflower oil; FL, 2% flaxseed oil; FI, 2% fish oil; SED, standard errors of the difference.

a,b Means within a row lacking a common superscript differ significantly (p<0.05).

** Number of chicks hatched alive analyzed with χ^2^ test.

**Table 5:** The effect of different dietary omega-3 and omega-6 fatty acid sources of female breeders on growth performance and slaughter weight of progeny

Day	Parameters	Maternal diets oil sources				SED	p-value

		SO	SA	FL	FI		
7	Feed intake (g/chicks)	215.8	196.2	190.2	206.2	3.86	0.137
	Body weight (g/chicks)	144.5	150.03	149.1	148.4	2.40	0.517
	Feed conversion rate (g feed intake/g BWG)	1.50	1.32	1.28	1.39	0.02	0.078
14	Feed intake (g/chicks)	718.3	685.8	645.2	693.8	10.28	0.129
	Body weight (g/chicks)	455.7	457.2	454.6	464.9	5.96	0.853
	Feed conversion rate(g feed intake/g BWG)	1.58	1.51	1.42	1.49	0.02	0.171
21	Feed intake (g/chicks)	1,369	1,334	1,332	1,368	18.14	0.808
	Body weight (g/chicks)	867.9	866.0	880.2	888.6	9.70	0.736
	Feed conversion rate (g feed intake/g BWG)	1.58	1.54	1.51	1.54	0.02	0.729
28	Feed intake (g/chicks)	2,262	2,347	2,321	2,362	26.67	0.584
	Body weight (g/chicks)	1,579	1,570	1,571	1,563	13.38	0.992
	Feed conversion rate (g feed intake/g BWG)	1.43	1.50	1.48	1.51	0.01	0.393
35	Feed intake (g/chicks)	3,486	3,586	3,630	3,668	42.43	0.485
	Body weight (g/chicks)	2,397	2,415	2,375	2,416	29.05	0.955
	Feed conversion rate (g feed intake/g BWG)	1.46	1.49	1.53	1.52	0.01	0.505
Livability (%)	Male	88.55	91.23	88.99	91.00	1.89	0.940
	Female	89.69	85.54	91.29	89.37	3.09	0.924
At slaughter	Live weight (g/bird)						
	Male	2,644	2,622	2,614	2,621	27.56	0.986
	Female	2,355	2,330	2,279	2,334	19.91	0.634
	Average	2,515	2,466	2,429	2,470	23.58	0.703

SO, 2% soybean oil; SA, 2% sunflower oil; FL, 2% flaxseed oil; FI, 2% fish oil; SED, standard errors of the difference; BWG, body weight gain.

**Table 6:** The effect of different dietary omega-3 and omega-6 fatty acid sources of parents on carcass parameters of progeny

Parameters	Gender	Maternal diets oil sources				SED	p-value

		SO	SA	FL	FI		
Body weight (g/chicks)	Male	2,745	2,756	2,740	2,820	16.79	0.304
	Female	2,351	2,413	2,362	2,387	10.22	0.147
	Average	2,548	2,584	2,551	2,607	17.01	0.559
Hot carcass weight (g/chicks)	Male	1,917	1,923	1,930	1,974	12.69	0.388
	Female	1,645	1,695	1,652	1,679	7.82	0.093
	Average	1,781	1,809	1,791	1,795	15.23	0.931
Abdominal fat (g/chicks)	Male	21.09	22.88	24.46	25.11	0.76	0.255
	Female	18.23	15.36	15.49	15.28	0.71	0.112
	Average	19.66	19.12	19.98	20.05	0.53	0.926
Abdominal fat (%)	Male	1.09	1.18	1.25	1.25	0.03	0.351
	Female	1.10	0.91	0.94	0.90	0.02	0.067
	Average	1.10	1.04	1.10	3.61	0.62	0.383
Carcass yield (%)	Male	69.78	69.81	70.43	69.98	0.15	0.421
	Female	69.97	70.22	69.96	70.30	0.16	0.840
	Average	69.88	70.02	70.20	68.72	0.36	0.489

SO, 2% soybean oil; SA, 2% sunflower oil; FL, 2% flaxseed oil; FI, 2% fish oil; SED, standard errors of the difference.
